# Supplementary material for: Correlation of fluorescence microscopy, electron microscopy, and NanoSIMS stable isotope imaging on a single tissue section
Source: Commun Biol. 2020 Jul 9;3:362. doi: 10.1038/s42003-020-1095-x (PMC7347930; doi:10.1038/s42003-020-1095-x)
Supplement: Supplementary file 1 — Supplementary Information [file 42003_2020_1095_MOESM1_ESM.pdf]

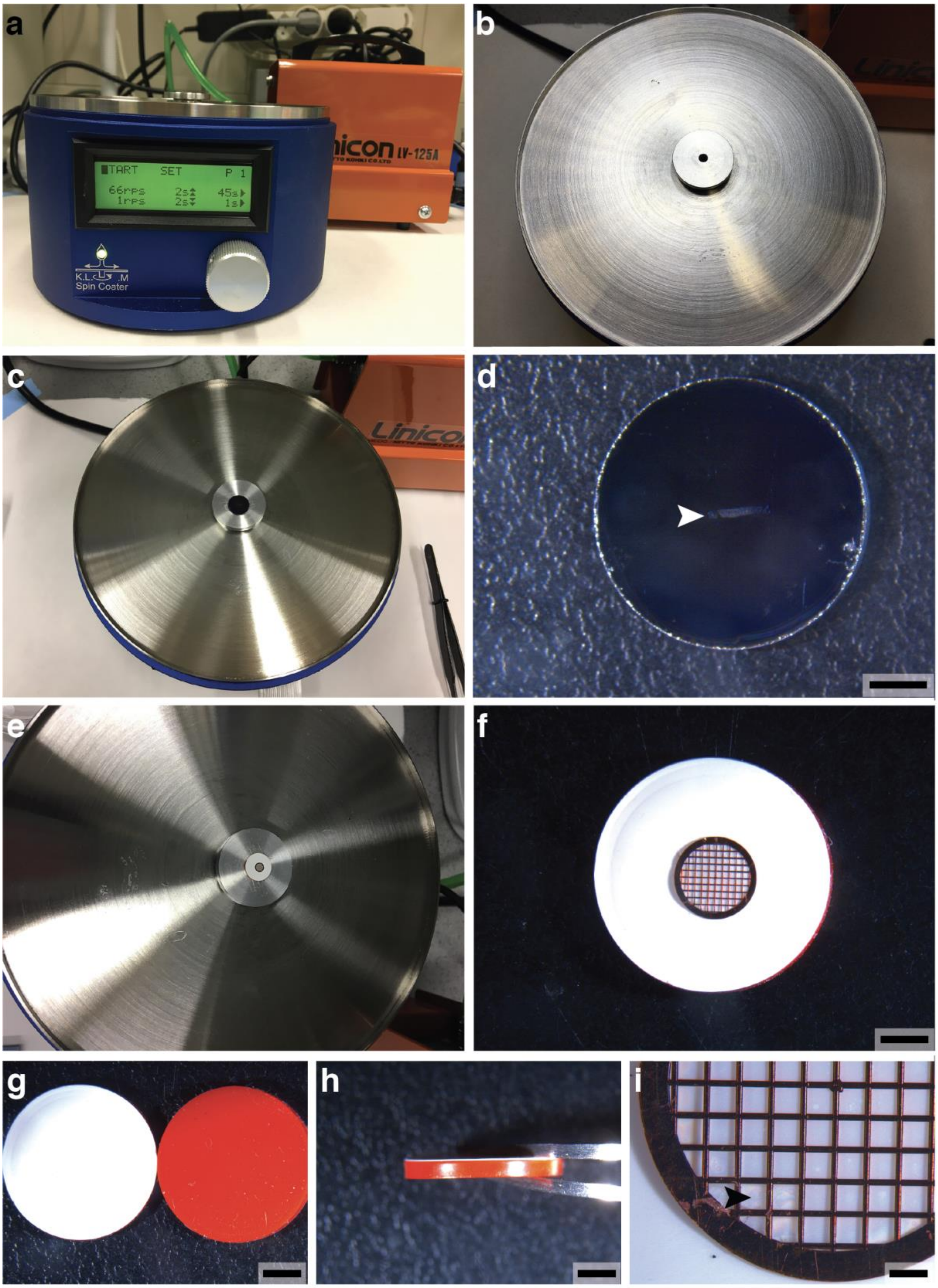

Supplementary Figure 1. The drying step of the cryosection with a PVA solution as embedding medium involves a tabletop spin dryer machine attached to a vacuum pump (A), which

generates enough vacuum to hold the sample in place over a 2 mm aperture in the center of the disk (B). NanoSIMS compatible holders (e.g., a 1 cm diameter coverslip or silicon wafer) can thus be directly placed over this aperture (C) for the drying step of the cryosection: White arrowhead in D points to a dried cryo section on a 1 cm diameter silicon wafer. If a TEM grid is used to hold the cryo section, a PTFE /rubber septa has to be used as support (E and F). This support is composed of two layers with one side (the PTFE; white in G and H) providing a non-adhesive and hydrophobic surface on which the TEM grids were placed. The other side (red in G and H) is made of rubber and provides perfect grip for the vacuum sealing. The integrity of the PVA film (black arrowhead in I) after spin drying can be controlled by simple visual inspection (depending on the eyesight of the operator). Scale bars: D, F, G and H: 2.5 mm and I: 200 μm.

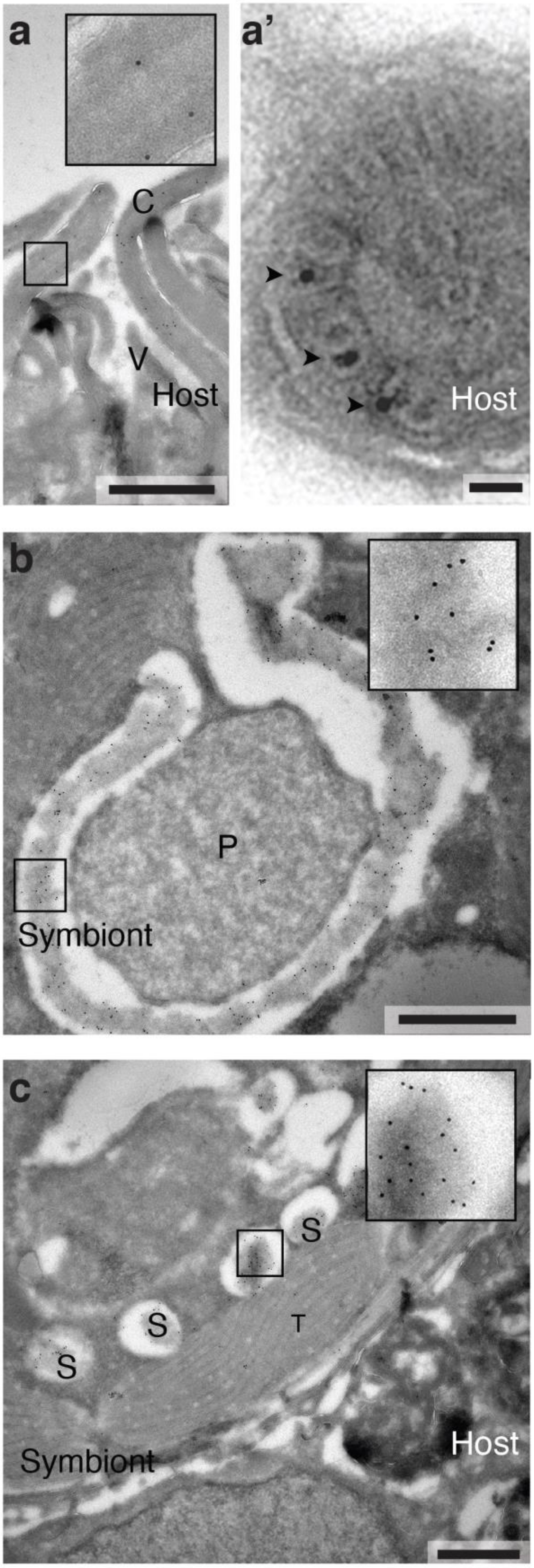
a
C
V
Host
a'
Host
b
P
Symbiont
c
S
S
S
T
Symbiont
Host

Supplementary Figure 2. The localization of the polyclonal antibody raised against β-tubulin was revealed by a secondary antibody linked to 10 nm gold particles (classical Tokuyasu sample preparation was used for the micrographs). Polymerized β-tubulin forms microtubules, which is a major component of the eukaryotic cytoskeleton. By immunolabelling, the antibody was observed inside the host cilia (A), in a ring of the outer doublet microtubules (black arrowheads in A'); villi were not labeled. The non-membrane delimited structure inside the pyrenoid (B) and the starch granules (C) were also immunolabeled. C: cilia; V: villi; T: thylakoid; P: pyrenoid; S: starch granule. Scale bars: 500 nm, except A' scale bar: 50 nm.

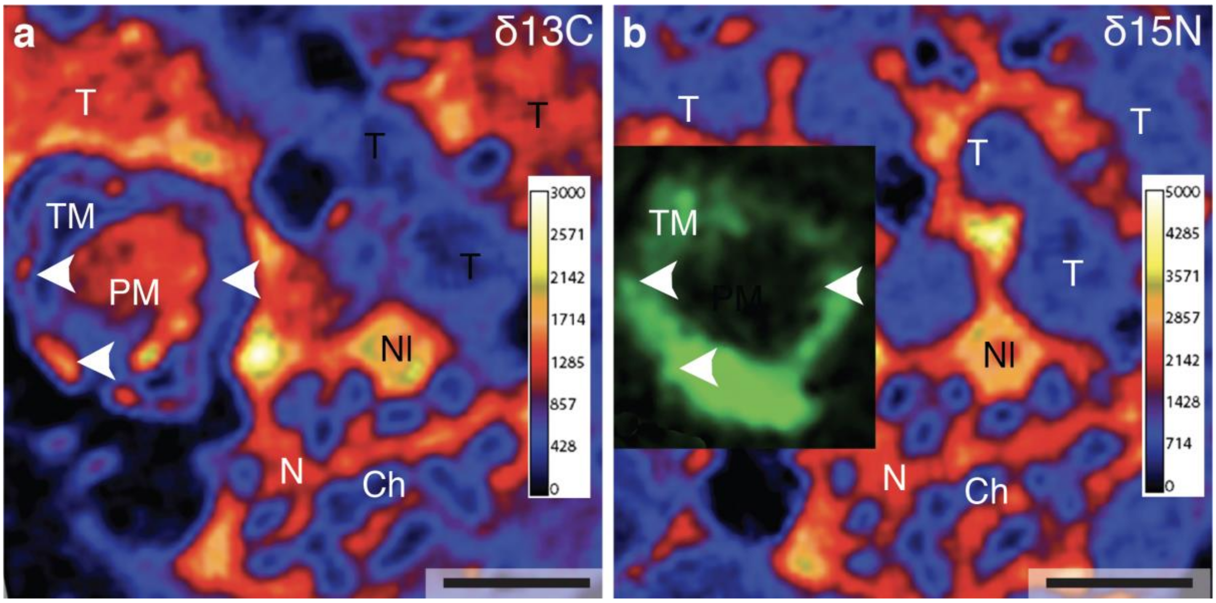

Supplementary Figure 3. High resolution overlays of the fluorescence image and TEM micrograph with the NanoSIMS isotopic enrichment map of ^{13}C (A) and ^{15}N (B) presented in Fig. 3. High enrichment levels of ^{13}C and ^{15}N were observed inside the dinoflagellate nucleus (except in the condensed chromosomes). The thylakoids and the tubule-like structure surrounding the pyrenoid matrix (black arrowheads), which also contained CA (green signal) and β-tubulin proteins (10 nm gold particles), were enriched in ^{13}C. The pyrenoid matrix exhibited both ^{13}C and ^{15}N enrichments, consistent with this structure being an active site of CO_2 fixation and a high rate of protein turn over. PM: pyrenoid matrix; N: nucleus; T: thylakoid; Nl: nucleolus; Ch: chromosome; TM: tubule-like matrix. Scale bars: A and B: 2 μm.

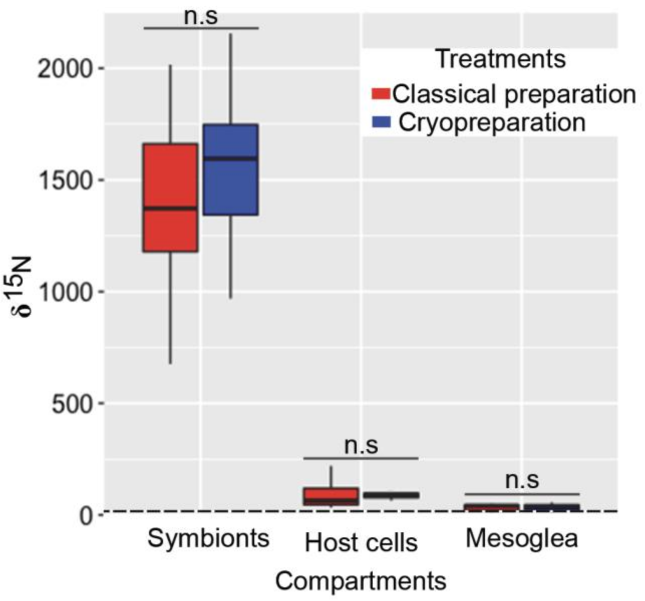

Supplementary Figure 4. Qualitative comparison of ionization efficiency between classically prepared (resin embedding) and the cryo preparation protocol (this method); chemical fixation was 2.5 % glutaraldehyde and 4 % formaldehyde in both preparations. The count-rate per second (cps) of $^{12}C^{14}N^{-}$ from coral tissue containing symbionts was a factor of ca. 1.8 times higher with our method. The statistical difference between the treatments was calculated by one-way ANOVA. The annotation of p-value significance level is: “***” [0, 0.001]; “**” (0.001, 0.01]; “*” (0.01, 0.05]; “.” (0.5, 0.1]; “ ” (0.1, 1]; “ns” non-significant.

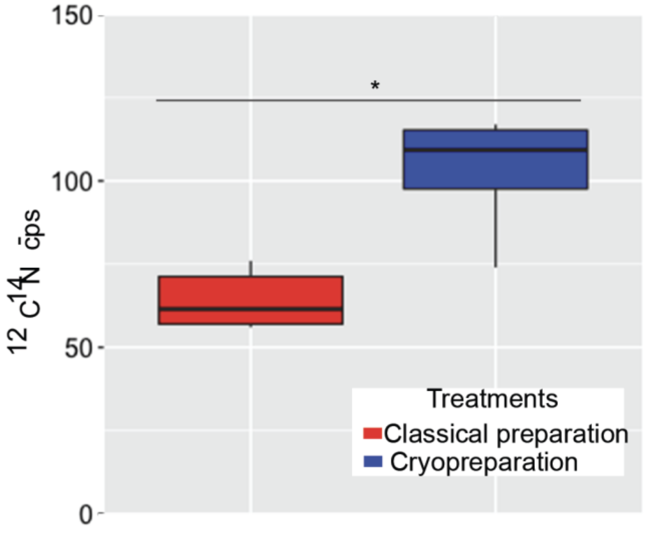

Supplementary Figure 5. Qualitative comparison of nitrate assimilation in a *S. pistillata* colony classically prepared (i.e., resin embedded) and with our method; chemical fixation was 2.5 % glutaraldehyde and 4 % formaldehyde in both preparations. The two sample preparation protocols yielded statistically indistinguishable ^{15}N enrichments for the three compartments (symbiont, host cells, and mesoglea). The dashed line indicates the detection limit for the NanoSIMS, defined here as 3 standard deviations obtained by analyzing similar regions in unlabeled control tissue (n = 9); The statistical difference between the treatments was calculated by one-way ANOVA. The annotation of p-value significance level is: "***" [0, 0.001]; "**" (0.001, 0.01]; "*" (0.01, 0.05]; "." (0.5, 0.1]; " " (0.1, 1]; "ns" non-significant.
