# Supplementary material for: Correlation of fluorescence microscopy, electron microscopy, and NanoSIMS stable isotope imaging on a single tissue section
Source: Commun Biol. 2020 Jul 9;3:362. doi: 10.1038/s42003-020-1095-x (PMC7347930; doi:10.1038/s42003-020-1095-x)
Supplement: Supplementary file 2 — Description of Additional Supplementary Files [file 42003_2020_1095_MOESM2_ESM.pdf]

Description of Additional Supplementary Items

Supplementary Data 1 (excel): Source data
