# Supplementary material for: Correlation of fluorescence microscopy, electron microscopy, and NanoSIMS stable isotope imaging on a single tissue section
Source: Commun Biol. 2020 Jul 9;3:362. doi: 10.1038/s42003-020-1095-x (PMC7347930; doi:10.1038/s42003-020-1095-x)
Supplement: Supplementary file 4 — Reporting Summary [file 42003_2020_1095_MOESM4_ESM.pdf]

nature research

Corresponding author(s): Celine Loussert-Fonta

Last updated by author(s): Jun 12, 2020

Reporting Summary

Nature Research wishes to improve the reproducibility of the work that we publish. This form provides structure for consistency and transparency in reporting. For further information on Nature Research policies, see our Editorial Policies and the Editorial Policy Checklist.

Statistics

For all statistical analyses, confirm that the following items are present in the figure legend, table legend, main text, or Methods section.

n/a	Confirmed	
☐	☒	The exact sample size (*n*) for each experimental group/condition, given as a discrete number and unit of measurement
☐	☒	A statement on whether measurements were taken from distinct samples or whether the same sample was measured repeatedly
☐	☒	The statistical test(s) used AND whether they are one- or two-sided<br>*Only common tests should be described solely by name; describe more complex techniques in the Methods section.*
☒	☐	A description of all covariates tested
☒	☐	A description of any assumptions or corrections, such as tests of normality and adjustment for multiple comparisons
☒	☐	A full description of the statistical parameters including central tendency (e.g. means) or other basic estimates (e.g. regression coefficient) AND variation (e.g. standard deviation) or associated estimates of uncertainty (e.g. confidence intervals)
☒	☐	For null hypothesis testing, the test statistic (e.g. F, t, r) with confidence intervals, effect sizes, degrees of freedom and P value noted<br>*Give P values as exact values whenever suitable.*
☒	☐	For Bayesian analysis, information on the choice of priors and Markov chain Monte Carlo settings
☒	☐	For hierarchical and complex designs, identification of the appropriate level for tests and full reporting of outcomes
☒	☐	Estimates of effect sizes (e.g. Cohen's d, Pearson's r), indicating how they were calculated

Our web collection on statistics for biologists contains articles on many of the points above.

Software and code

Policy information about availability of computer code

Data collection	No software was used
Data analysis	No software was used

For manuscripts utilizing custom algorithms or software that are central to the research but not yet described in published literature, software must be made available to editors and reviewers. We strongly encourage code deposition in a community repository (e.g. GitHub). See the Nature Research guidelines for submitting code & software for further information.

Data

Policy information about availability of data

All manuscripts must include a data availability statement. This statement should provide the following information, where applicable:

- Accession codes, unique identifiers, or web links for publicly available datasets
- A list of figures that have associated raw data
- A description of any restrictions on data availability

The data that support the findings of this study are available from the corresponding author upon reasonable request.

Field-specific reporting

Life sciences study design

All studies must disclose on these points even when the disclosure is negative.

Sample size	Three different mother colonies were selected according the similarity of their sizes
Data exclusions	No data were excluded.
Replication	All attemps of replication were successful
Randomization	This is no relevant to this sudy as we are comparing 2 methods of sample preparation.
Blinding	This is no relevant to this sudy as we are comparing 2 methods of sample preparation.

Reporting for specific materials, systems and methods

We require information from authors about some types of materials, experimental systems and methods used in many studies. Here, indicate whether each material, system or method listed is relevant to your study. If you are not sure if a list item applies to your research, read the appropriate section before selecting a response.

Materials & experimental systems

n/a	Involved in the study	
☐	☒	Antibodies
☒	☐	Eukaryotic cell lines
☒	☐	Palaeontology and archaeology
☐	☒	Animals and other organisms
☒	☐	Human research participants
☒	☐	Clinical data
☒	☐	Dual use research of concern

Methods

n/a	Involved in the study	
☒	☐	ChIP-seq
☒	☐	Flow cytometry
☒	☐	MRI-based neuroimaging

Antibodies

Antibodies used	mouse anti-beta-tubulin antibody (Sigma-Aldrich, Switzerland), rabbit anti-carbonic anhydrase (reference 100-4157, Rockland, US), donkey anti-rabbit linked with Alexa 568 (ThermoFisher, US), 10 nm colloidal gold-conjugated secondary antibodies, goat anti-mouse (Aurion, The Netherlands)
Validation	Antibodies validation was done according Griffiths G, Lucocq JM. Antibodies for immunolabeling by light and electron microscopy: not for the faint hearted. Histochem Cell Biol. 2014;142(4):347-360. doi:10.1007/s00418-014-1263-5.

Animals and other organisms

Policy information about studies involving animals; ARRIVE guidelines recommended for reporting animal research

Laboratory animals	The study did not involve laboratory animals
Wild animals	The study did not involve wild animals
Field-collected samples	Specimens of Stylophora pistillata were collected from the shallow waters (4–8 m) of the Gulf of Aqaba in Eilat, Israel under permit 2019/42143 of the Israel Nature and Parks Authority. Experiments were conducted in the outdoor Red Sea Simulator system at the Interuniversity Institute (IUI) for Marine Sciences with corals maintained in air-bubbled aquaria (2 L) at ambient water temperature (28 °C) and reduced solar irradiance (ca. 200 µmol m-2 sec -1 at midday under canopies).
Ethics oversight	Permit 2019/42143 of the Israel Nature and Parks Authority

Note that full information on the approval of the study protocol must also be provided in the manuscript.
